# Quantification of Unencapsulated Drug in Target Tissues Demonstrates Pharmacological Properties and Therapeutic Effects of Liposomal Topotecan (FF-10850)

**DOI:** 10.1007/s11095-023-03652-4

**Published:** 2024-03-27

**Authors:** Toshifumi Kimura, Ken Okada, Yasushi Morohashi, Shun Matsuda, Yukio Kato, Mikinaga Mori, Hiroshi Kato, Takeshi Matsumoto, Susumu Shimoyama

**Affiliations:** 1 Bio Science & Engineering Laboratories, FUJIFILM Corporation, 577 Ushijima, Kaisei-Machi, Ashigarakami-Gun, Kanagawa, 258-8577 Japan; 2 Faculty of Pharmacy, Kanazawa University, Kakuma-Machi, Kanazawa, Ishikawa 920-1192 Japan; 3 FUJIFILM Pharmaceuticals U.S.A., Inc, One Broadway, Cambridge, MA 02142 USA; 4 Safety Evaluation Center, Ecology & Quality Management Division, CSR Division, FUJIFILM Corporation, 210 Nakanuma, Minamiashigara, 250-0193 Kanagawa Japan

**Keywords:** drug release, liposome, ovarian cancer, tissue distribution, topotecan

## Abstract

**Purpose:**

Quantifying unencapsulated drug concentrations in tissues is crucial for understanding the mechanisms underlying the efficacy and safety of liposomal drugs; however, the methodology for this has not been fully established. Herein, we aimed to investigate the enhanced therapeutic potential of a pegylated liposomal formulation of topotecan (FF-10850) by analyzing the concentrations of the unencapsulated drug in target tissues, to guide the improvement of its dosing regimen.

**Methods:**

We developed a method for measuring unencapsulated topotecan concentrations in tumor and bone marrow interstitial fluid (BM-ISF) and applied this method to pharmacokinetic assessments. The ratios of the area under the concentration–time curves (AUCs) between tumor and BM-ISF were calculated for total and unencapsulated topotecan. DNA damage and antitumor effects of FF-10850 or non-liposomal topotecan (TPT) were evaluated in an ES-2 mice xenograft model.

**Results:**

FF-10850 exhibited a much larger AUC ratio between tumor and BM-ISF for unencapsulated topotecan (2.96), but not for total topotecan (0.752), than TPT (0.833). FF-10850 promoted milder DNA damage in the bone marrow than TPT; however, FF-10850 and TPT elicited comparable DNA damage in the tumor. These findings highlight the greater tumor exposure to unencapsulated topotecan and lower bone marrow exposure to FF-10850 than TPT. The dosing regimen was successfully improved based on the kinetics of unencapsulated topotecan and DNA damage.

**Conclusions:**

Tissue pharmacokinetics of unencapsulated topotecan elucidated the favorable pharmacological properties of FF-10850. Evaluation of tissue exposure to an unencapsulated drug with appropriate pharmacodynamic markers can be valuable in optimizing liposomal drugs and dosing regimens.

**Supplementary Information:**

The online version contains supplementary material available at https://doi.org/10.1007/s11095-023-03652-4.

## Introduction

Liposomal drugs encapsulating low molecular weight compounds are recognized as beneficial pharmaceuticals that improve solubility, circulating half-life, and drug delivery efficiency to the target site [1–5]. In cancer treatment, the accumulation of liposomes in tumor tissues, known as enhanced permeabilization and retention (EPR), is facilitated by leaky tumor blood vessels and impaired lymphatic drainage. This phenomenon increases the effectiveness of the long-circulating liposomal antitumor drugs [6–9]. Pegylated liposomal doxorubicin (Doxil/Caelyx®) was the first anticancer liposomal therapeutic designed to exploit the EPR effect and was approved by FDA in 1995 based on its superior safety and equivalent efficacy to conventional non-liposomal doxorubicin [10]. In patients, systemic doxorubicin exposure after injection of Doxil/Caelyx® was found to be nearly 300-fold greater than that of conventional non-liposomal doxorubicin [8]. However, the encapsulated drug is biologically unavailable and must be released to exert its biological activities. Therefore, in addition to the deposition of liposomes in the tumor tissue, sustained and/or stimulus-triggered payload release in the tumor microenvironment is critical to improve the therapeutic window when compared with conventional drugs [11–13].

Pharmacokinetic-pharmacodynamic (PK/PD) modeling and simulation is one of the most powerful tools for quantitatively understanding critical factors that influence the deposition of liposomal drugs and payload release profiles. Limited studies have addressed the modeling of bioavailable drug exposure at target sites for liposomal therapeutics [14]. An important challenge in improving the PK/PD modeling approach is obtaining quantitative information regarding active drug concentrations at the target sites [15]. While most pharmacokinetic and biodistribution studies of liposomal anticancer drugs have quantified total drug concentration (sum of the encapsulated and unencapsulated), only a few studies have analyzed the unencapsulated drug in plasma [4, 16]. However, these studies provided minimal data regarding the local bioavailable drug exposure, specifically the unencapsulated drug concentrations in tumor and normal tissues. Technical difficulties in quantifying unencapsulated drugs in solid tissues, owing to potential overestimation due to liposome rupture during tissue homogenization and insufficient recovery rates, present challenges for assessing tissue pharmacokinetics [17]. A universally applicable methodology for the quantification of unencapsulated drugs is yet to be established.

Topotecan (TPT), an approved chemotherapeutic agent for treating ovarian cancer, small-cell lung cancer, and cervical cancer via intravenous infusion [18], exhibits its tumor inhibitory effect in a time and concentration-dependent manner [19, 20]. However, it is rapidly cleared from circulation mainly via urinary and fecal excretion [21, 22], limiting its therapeutic outcomes. Another major clinical issue of topotecan is treatment discontinuation or dose reduction owing to severe myelosuppression [23]. Topotecan exerts its cytotoxic effect by binding to DNA topoisomerase I, causing single- and double-strand DNA breaks and subsequent cell death [24, 25]. Considering its cytotoxicity, particularly on cells in the S-phase, topotecan damages rapidly renewing healthy tissues such as hematopoietic cells. To overcome these limitations, FF-10850, a pegylated topotecan liposomal injection, approximately 110 nm in diameter, with encapsulation efficiency exceeding 99.8%, and storage stability for 24 months under refrigeration, has been recently developed [26]. FF-10850 stably encapsulates topotecan within the dihydrosphingomyelin-based liposomal shell and has demonstrated prolonged plasma circulating time, greater antitumor activity, and milder hematological toxicities than the non-liposomal TPT in mice [26, 27] and is currently under clinical investigation (NCT04047251). However, the exposure of tissues to unencapsulated topotecan remains poorly clarified. In the current study, we aimed to elucidate the differences in pharmacological effects between FF-10850 and non-liposomal TPT through tissue pharmacokinetic analysis. To investigate the unencapsulated drug exposure and the efficacy and toxicity at target tissues, we developed methods to separate unencapsulated from encapsulated topotecan and quantify unencapsulated topotecan concentrations in the tumor tissue and bone marrow interstitial fluid (BM-ISF). Additionally, we attempted to improve the dosing schedule of FF-10850 based on tissue pharmacokinetics and kinetics of a pharmacodynamic marker following FF-10850 administration.

## Materials and Methods

### Materials

FF-10850 was prepared according to a previously described method [26]. Topotecan was purchased from ScinoPharm Taiwan (Tainan, Taiwan) and topotecan-d6 was purchased from Toronto Research Chemicals (Toronto, Canada). Phosphate buffered saline (PBS), methanol, and formic acid were purchased from Wako Pure Chemical Industries (Osaka, Japan).

### Animals

Female BALB/c nude mice (5 weeks old) were purchased from CLEA Japan (Tokyo, Japan). The animal study protocols were approved by the Institutional Animal Care and Use Committee of FUJIFILM Corporation, and the experiments were conducted in compliance with the Act on Welfare and Management of Animals and Code of Welfare of Laboratory Animals of FUJIFILM Corporation. The animals were housed under controlled temperature, humidity, and a dark–light cycle, with *ad libitum* access to water and diet. Animals without abnormalities in clinical signs or body weight during the quarantine and acclimation periods were used for studies.

### Blood and Tissue Sampling for Pharmacokinetic Studies

ES-2 tumor bearing mice received a single intravenous injection of 2 mg/kg of topotecan, either as FF-10850 or TPT, via the tail vein. Sucrose/histidine buffer and saline (Otsuka Pharmaceutical Factory, Tokushima, Japan) were used as the diluent for FF-10850 and TPT, respectively. After blood sample collection, mice were euthanized at the corresponding time points, and tumor and bone marrow tissues were collected. Plasma was obtained by centrifuging the blood samples at 800 × *g* for 15 min at 4 °C. Tumor tissues, placed on ice, were minced into fine pieces with scissors. Bone marrow tissues were harvested from the femur and tibia. After cutting the ends, the bones were placed in a microtube and centrifuged at 1,200 × *g* for 1 min at 4 °C. Unencapsulated topotecan was immediately separated from the plasma and tissue samples and processed.

### Separation of Unencapsulated Topotecan

For the separation of unencapsulated topotecan in plasma, plasma samples diluted with PBS were ultracentrifuged at 200,000 × *g* for 1 h at 4 °C to precipitate the liposomes. The supernatants were collected as samples for measuring unencapsulated topotecan in plasma. To separate unencapsulated topotecan in tumor tissues, the tissues were minced as described and homogenized in PBS containing topotecan-d6 as an internal standard (125 mg tissue/mL PBS), with zirconia balls using Micro Smash MS-100R (Tomy Seiko, Tokyo, Japan). The homogenate was then ultracentrifuged at 200,000 × *g* for 1 h at 4 °C, and the supernatants were collected as samples for measuring unencapsulated topotecan in the tumor tissue. For the separation of unencapsulated topotecan in BM-ISF, a bone marrow tissue suspension was prepared by adding PBS containing topoteacan-d6 to the tissue (25 mg tissue/mL PBS) and suspending it by pipetting. After removing cellular components from the suspension by centrifugation at 1,200 × *g* for 3 min at 4 °C, the supernatant was collected as the BM-ISF sample. These samples were ultracentrifuged at 200,000 × *g* for 1 h at 4 °C, and the supernatants were collected to measure unencapsulated topotecan in the BM-ISF.

To determine whether FF-10850 remained intact during tissue homogenization and separation processes in tumor and bone marrow tissues, unencapsulated topotecan concentrations were measured in samples spiked with FF-10850. Tumor tissues from untreated mice were spiked with FF-10850 to obtain homogenates containing 250, 1,250, or 5,000 ng/mL of total topotecan. Spiked tumor samples were homogenized according to the procedure described above. For comparison, homogenates were prepared using the conventional tissue homogenization method, with spiked tumor tissue samples frozen in liquid nitrogen and subjected to pulverization by multi-beads shocker (Yasui Kikai, Osaka, Japan) with metal corn. Bone marrow tissue suspensions from untreated mice were spiked with FF-10850 (6,033 ng/mL as total topotecan) plus non-liposomal topotecan (0, 10, 200, or 5,000 ng/mL).

### Quantification of Topotecan

To prepare the samples, methanol with 0.1% formic acid plus topotecan-d6 was added to plasma samples, while methanol with 0.1% formic acid was used for tumor and BM-ISF samples. The samples were thoroughly mixed and centrifuged at 11,300 × *g* for 3 min. The supernatants were added to an equal volume of water with 0.1% formic acid, and the mixture was injected into the liquid chromatographic–triple quadrupole mass spectrometric (LC–MS/MS) system. The ultra-performance liquid chromatography (UPLC) conditions for topotecan analysis were as follows: system, ACQUITY UPLC (Waters, Milford, MA); column, ACQUITY UPLC BEH C18 Column (1.7 µm, 2.1 mm × 50 mm, Waters); mobile phase A, 0.1% formic acid; mobile phase B, methanol with 0.1% formic acid, gradient (initial to 2.0 min, linear increase from 20 to 50% of B; 2.0 to 3.0 min, 95% B, and 3.0 to 4.0 min, 20% B); flow rate, 0.4 mL/min; column temperature, 40 °C. A Triple Quad 5500 (AB Sciex, Framingham, MA) mass spectrometer equipped with Analyst software (AB Sciex) and positive ion electrospray ionization in a multiple reaction monitoring (MRM) mode were used. Quantification was performed using MRM with the transitions of m/z at 422.0 → 377.1 for topotecan and 428.1 → 377.1 for topotecan-d6.

### Pharmacokinetic Analysis

Pharmacokinetic parameters, including half-life (T_1/2_), initial concentration at t = 0 (C_0_), the area under the concentration *versus* time curves from 0 to infinity (AUC_inf_), clearance (CL), mean residence time (MRT), and the volume of distribution (Vd) of total and unencapsulated topotecan in plasma, tumor tissue, and BM-ISF were analyzed using noncompartmental analysis with Phoenix WinNonlin version 8.2 pharmacokinetic software (Certara, Princeton, NJ). The AUCs were calculated using the linear up/log down rule.

### Assessment of DNA Damage

ES-2 tumor-bearing mice received a single intravenous administration of 2 mg/kg of FF-10850 or repeated administration of TPT for 5 consecutive days at a dose of 2 mg/kg/day; this is compatible with clinically approved dosing schedule, specifically daily dosing on day 1 to 5 of each 21-day cycle. The dose of TPT was determined as the maximum tolerated dose in mice based on our previous study (data not shown). Mice in the FF-10850 group were euthanized at 0.5, 3, and 24 h, and 3, 5, 7, 9, and 11 days post-dose (*n* = 3). Mice in the TPT group were euthanized at 3 and 24 h after each dosing and 3, 5, and 7 days after the last dosing (*n* = 3). Samples were also harvested at 0.5 h after the first and last TPT dose (*n* = 3). Mice in the vehicle control group were administered sucrose/histidine buffer intravenously and were euthanized at 3 h, 3, 7, and 11 days post-dosing (*n* = 2 or 3). The levels of Ser139-phosphorylated human H2AX (γhH2AX) and total hH2AX in the tumor, as well as Ser139-phosphorylated mouse H2AX (γmH2AX) and total mH2AX in mouse bone marrow, were quantified using a previously established method [28].

### Assessment of Antitumor Effect in Mouse Xenograft Model

The human ovarian cancer cell line ES-2 was obtained from ATCC (Manassas, VA). ES-2 cells were cultured in McCoy’s 5A medium (Thermo Fisher Scientific, Waltham, MA) supplemented with 10% FBS and 100 U/mL penicillin and 100 mg/mL streptomycin (Thermo Fisher Scientific, Waltham, MA) at 37 °C under 5% CO_2_, and passaged every 3 or 4 days. ES-2 cells suspended in 0.1 mL McCoy’s 5A at a density of 3 × 10^7^ cells/mL were subcutaneously injected into the side flank of BALB/c nude mice. Seven days after inoculation, tumor width and length were measured, and tumor volume was calculated as (width)^2^ × (length) × 0.5. Mice were randomized (*n* = 8/group) into 5 groups: control (vehicle), control (empty liposome), FF-10850 single dose, FF-10850 weekly dose, and TPT, using randomized block method by StatLight (Yukms, Kanagawa, Japan) based on tumor volumes. Briefly, mice in the FF-10850 treatment group were administered either a single intravenous dose of 2 mg/kg FF-10850 or a once-weekly dose of 1 mg/kg/week FF-10850 for 2 weeks via tail vein. Mice in the TPT treatment group received 2 mg/kg of TPT for five consecutive days. Mice in the vehicle control groups were administered intravenously sucrose/histidine buffer, the diluent for FF-10850, or empty liposomes every week. Tumor volume and body weight were measured twice weekly.

### Statistical Analysis

Statistical analysis of tumor volume was performed using Bartlett’s test followed by the Steel–Dwass test using the JMP software (SAS Institute, Cary, NC). P values < 0.01 were considered statistically significant.

## Results

### Pharmacokinetics of Total Topotecan after Administration of FF-10850 and TPT

**Fig. 1:**
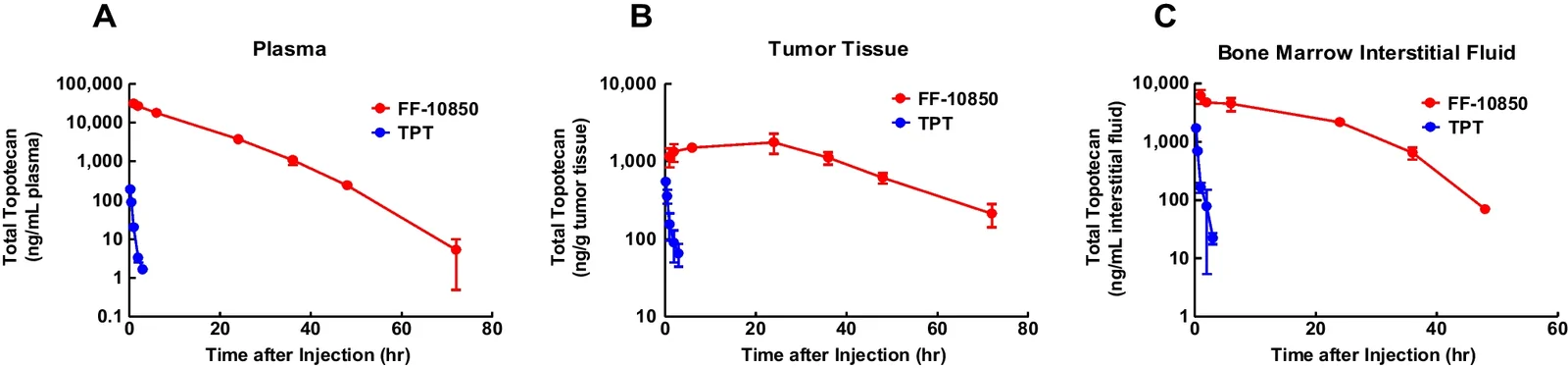
Total topotecan concentration in ES-2 xenograft model mice receiving FF-10850 or TPT. ES-2 xenograft model mice were intravenously injected with 2 mg/kg of FF-10850 or TPT. The concentrations of total topotecan in plasma (**A**), tumor tissues (**B**), and BM-ISF (**C**) were determined at various time points after administration: 1, 2, 6, 24, 36, 48, and 72 h for FF-10850, and 0.25, 0.5, 1, 2, and 3 h for TPT. Concentrations below the lower limit of quantification were not shown. Data are presented as mean ± standard deviation (*n* = 3).

In our recent study [27], we observed that FF-10850 exhibited superior antitumor activity and mitigated hematological toxicities when compared with TPT. To gain insights into the pharmacokinetic behavior of FF-10850 underlying these favorable outcomes, we investigated tissue distribution of total topotecan in plasma, tumor, and BM-ISF. Total topotecan concentrations were measured following a single administration of FF-10850 and TPT at a dosage of 2 mg/kg in ES-2 tumor-bearing mice (Fig. 1A-C). Following intravenous administration of TPT, the concentrations of topotecan in plasma, tumor, and BM-ISF rapidly declined. In contrast, the tumor concentration of total topotecan following administration of FF-10850 increased initially up to 24 h post-dose, followed by a gradual decrease (Fig. 1B). This pattern suggests the accumulation and retention of liposomes in the tumor. Table I summarizes the pharmacokinetic parameters. The slower elimination of total topotecan after FF-10850 administration was reflected by lower CL and prolonged T_1/2_ and MRT (Table I). Consistent with previous reports [29], topotecan exhibited high distribution in BM-ISF after TPT administration, with the AUC_inf_ in BM-ISF being 6 times greater than that in plasma (Table I). The AUC_inf_ ratios of total topotecan between the tumor and BM-ISF were nearly comparable between FF-10850 and TPT (Table I). Therefore, the total topotecan exposure profile of FF-10850 was inconsistent with its superior pharmacological effects compared to TPT.

**Table I:** Pharmacokinetic parameters for total topotecan in plasma, tumor, and BM-ISF following intravenous injection of FF-10850 and TPT

Formulation		TPT	FF-10850
Plasma			
T_1/2_	h	0.399	4.63
C_0_	ng/mL	406	3.61 × 10^4^
AUC_inf_	ng∙h/mL	140	3.47 × 10^5^
CL	mL/h/kg	1.43 × 10^4^	5.77
MRT	h	0.388	10.3
Vd	mL/kg	5.87 × 10^3^	59.2
Tumor			
T_1/2_	h	0.928	15.1
C_max_	ng/g tissue	546	1.77 × 10^3^
AUC_inf_	ng∙h/g tissue	582	7.78 × 10^4^
MRT	h	1.02	26.5
BM-ISF^a)^			
T_1/2_	h	0.690	4.83
C_max_	ng/mL	1.71 × 10^3^	6.09 × 10^3^
AUC_inf_	ng∙h/mL	862	1.03 × 10^5^
MRT	h	0.659	14.1
AUC_inf_ ratio (Tumor/BM-ISF)		0.675	0.752

a) Bone marrow interstitial fluid.

### Method Development for Unencapsulated Topotecan Quantification

The intravenous injection of liposomal drugs often results in the majority of the drug being entrapped within liposomes, rendering it biologically inactive even despite a long half-life [30]. In our previous study [27], we hypothesized that the tumor exposure to the unencapsulated topotecan would be greater in the FF-10850 group than in the TPT group. We proposed that this preferential exposure in the tumor is mediated by payload release via tumor-associated macrophages and ammonia. Conversely, we expected lower bone marrow exposure to unencapsulated topotecan after FF-10850 injection when compared with TPT.

**Fig. 2:**
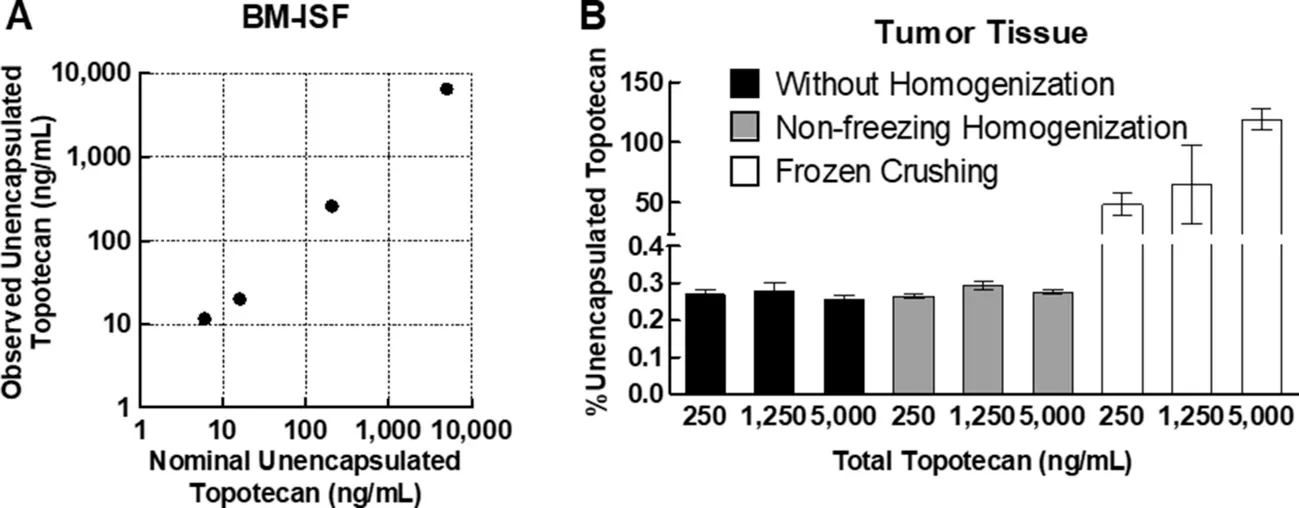
Separation method for total and unencapsulated topotecan from BM-ISF and tumor tissue. The method for separately measuring the total and unencapsulated topotecan in BM-ISF and tumor tissues was evaluated using *in vitro* spiked samples. (**A**) Bone marrow tissue suspension obtained from untreated mice was spiked with FF-10850 (6,033 ng/mL as total topotecan) and various concentrations of non-liposomal TPT, followed by the separate quantification of unencapsulated topotecan. The nominal concentrations of unencapsulated topotecan represent the sum of non-liposomal TPT spiked and unencapsulated topotecan present in the outer water phase of FF-10850. Data are presented as mean ± standard deviation (*n* = 3). (**B**) The integrity of FF-10850 assessed following the homogenization of tumor tissues. ES-2 tumor specimens obtained from untreated mice were spiked with various concentrations of FF-10850 and subjected to conventional frozen crushing or non-freezing homogenization developed in the current study. The total and unencapsulated topotecan were quantified, and as a control, total and unencapsulated topotecan in the tumor homogenate frozen crushed prior to spiking were also measured. The unencapsulated topotecan was normalized with total concentration and represented as mean ± standard deviation (*n* = 3).

To assess the unencapsulated topotecan exposure in tissue samples, we developed a procedure for separating the unencapsulated topotecan from tissue suspension and homogenate samples. Although a method for separating unencapsulated drug from plasma samples using ultracentrifugation had been reported previously [31], no such method exists for tissue samples. Accurate quantification requires a high recovery rate of unencapsulated topotecan from tissue samples and the minimization of topotecan leakage from liposomes during sample processing. Therefore, we examined the recovery and potential leakage of unencapsulated topotecan from liposomes using FF-10850 and non-liposomal topotecan spiked *in vitro* samples. Freshly prepared mouse bone marrow tissue suspension was spiked with FF-10850 (6,033 ng/mL as total topotecan, including approximately 6 ng/mL of unencapsulated topotecan present in the outer water phase of formulation) and 0–5,000 ng/mL of non-liposomal topotecan. We then separated the unencapsulated topotecan, observing that the measured concentrations were consistent with nominal values (Fig. 2A), indicating a successful removal of encapsulated topotecan and sufficient recovery of unencapsulated topotecan from the suspension samples.

Next, we focused on developing a homogenization method for solid tissues to quantify the unencapsulated topotecan. For frozen tissues, mechanical crushing methods are often used to prepare tissue homogenates; however, there is a great concern regarding liposome destabilization during the freeze-thawing process [32]. To minimize mechanical stress and prepare suspensions from non-frozen tumor tissue samples, the specimens were cut into small pieces prior to mechanical homogenization, as described in the Materials and Methods section. Figure 2B shows the amount of unencapsulated topotecan measured in the tumor homogenates spiked with FF-10850 before and after the homogenization process. The conventional frozen crushing method generated a large amount of unencapsulated topotecan, suggesting the destabilization of FF-10850 during the freeze-thawing process. Conversely, the homogenization method we developed maintained the unencapsulated topotecan concentration as low as that of the control homogenate (Fig. 2B). These observations indicate that FF-10850 remains intact during the sample preparation process in our developed homogenization method.

#### Pharmacokinetics of Unencapsulated Topotecan Following Administration of FF-10850 and TPT

**Fig. 3:**
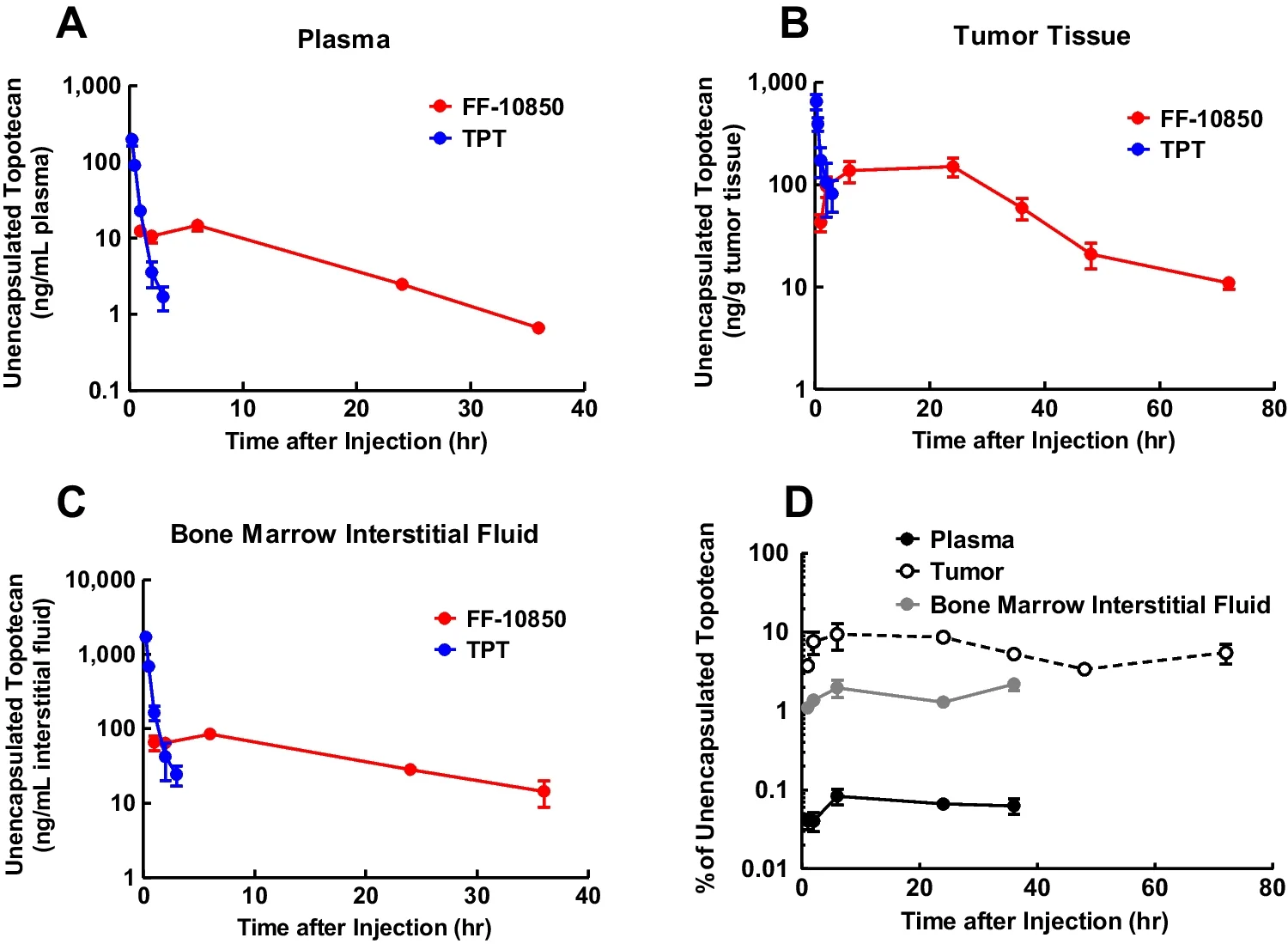
Unencapsulated topotecan concentrations in ES-2 xenograft model mice receiving FF-10850 or TPT. ES-2 xenograft model mice were injected with 2 mg/kg of FF-10850 or TPT. The concentrations of unencapsulated topotecan in plasma (**A**), ES-2 tumor tissue (**B**), and BM-ISF (**C**) were determined at various time points after administration: 1, 2, 6, 24, 36, 48, and 72 h for FF-10850, 0.25, 0.5, 1, 2, and 3 h for TPT. Concentrations below the lower limit of quantification are not shown. (**D**) Percentage of topotecan present in its unencapsulated form following FF-10850 injection. Data are presented as mean ± standard deviation (*n* = 3).

Figure 3A-C illustrates the pharmacokinetic profiles of unencapsulated topotecan in plasma, tumor tissue, and BM-ISF, respectively, in mice intravenously injected with FF-10850 or TPT. The time-concentration profiles of the unencapsulated topotecan after TPT administration closely resembled those of total topotecan (Fig. 1). This similarity suggests that unencapsulated topotecan was successfully extracted from the tissues. In contrast, the plasma peak concentration of unencapsulated topotecan after FF-10850 administration (Fig. 3A) was > 1,000-fold lower than that of total topotecan (Fig. 1A). This difference can be attributed to the stable encapsulation of the payload and rapid clearance of unencapsulated topotecan from circulation. Similarly, the peak concentrations of unencapsulated topotecan in tumor tissue and BM-ISF after FF-10850 administration (Fig. 3B and C) were remarkably lower than those of total topotecan (Fig. 1B and C). However, the concentration of unencapsulated topotecan in the tumor gradually increased 6 h post-dosing with FF-10850, followed by sustained levels until 24 h and then a gradual decrease (Fig. 3B), remaining measurable until the end of the sampling period. This contrasted with the rapid decrease in tumor unencapsulated topotecan concentration profile following TPT administration (Fig. 3B). In BM-ISF, unencapsulated topotecan reached C_max_ at 6 h post-dosing with FF-10850 and decreased below the detection limit (13.7 ng/mL, Fig. 3C). Figure 3D presents the percentage of topotecan in the unencapsulated form in plasma and tissues after FF-10850 injection. The fraction of unencapsulated topotecan in BM-ISF (1–3%) was higher than that in plasma (< 0.1%) but lower than that in tumor (3–10%) (Fig. 3D). Pharmacokinetic parameters of unencapsulated topotecan are summarized in Table II. For TPT, the difference between total and unencapsulated topotecan AUCs was within 1.5-fold in plasma and each tissue (Table I and II). Conversely, a 1,616-fold (plasma), 14.5-fold (tumor), and 56.9-fold (BM-ISF) smaller AUC_inf_ of unencapsulated topotecan relative to total topotecan were observed in the FF-10850 group (Table I and II). The AUC ratio of unencapsulated topotecan between tumor and BM-ISF was 0.833 for TPT, which was close to that of total topotecan, but 2.96 for FF-10850, which was larger than that of total topotecan (Table I and II). These results indicate that tumor cells were exposed to higher concentrations of unencapsulated topotecan compared to bone marrow cells after FF-10850 administration. This observation is consistent with improved therapeutic index and reduced hematotoxicity.

**Table II:** Pharmacokinetic parameters for unencapsulated topotecan in plasma, tumor, and BM-ISF following intravenous injection of FF-10850 and TPT

Formulation		TPT	FF-10850
Plasma			
T_1/2_	h	0.439	6.72
C_max_	ng/mL	198	14.7
AUC_inf_	ng∙h/mL	97.3	215
MRT	h	0.577	10.8
Tumor			
T_1/2_	h	0.971	15.8
C_max_	ng/g tissue	646	150
AUC_inf_	ng∙h/g tissue	684	5.38 × 10^3^
MRT	h	1.04	22.6
BM-ISF^a)^			
T_1/2_	h	0.729	11.7
C_max_	ng/mL	1.70 × 10^3^	85.0
AUC_inf_	ng∙h/mL	822	1.82 × 10^3^
MRT	h	0.607	13.4
AUC_inf_ ratio (Tumor/BM-ISF)		0.833	2.96

a) Bone marrow interstitial fluid.

#### Pharmacodynamic Responses to FF-10850 and TPT

**Fig. 4:**
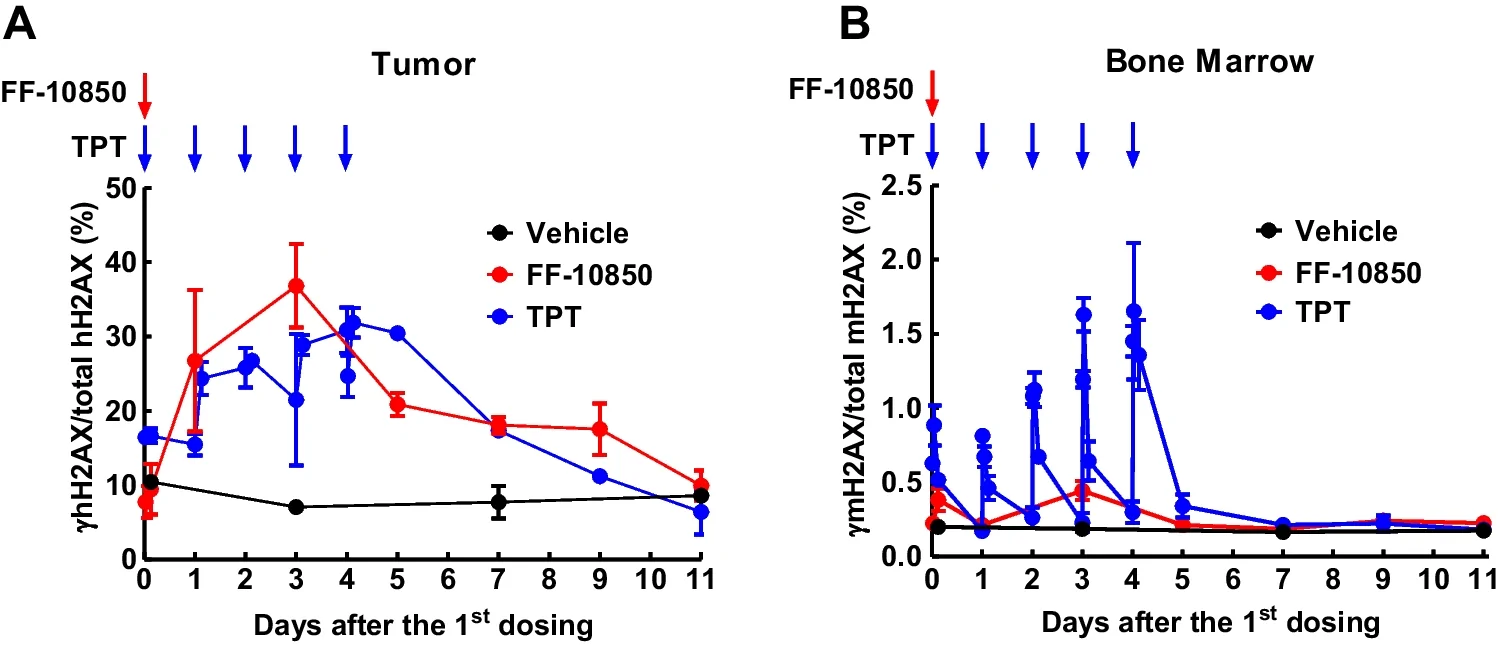
DNA damaging effects in tumor and bone marrow following treatment with FF-10850 or TPT. BALB/c nude mice were subcutaneously engrafted with the ES-2 human ovarian cancer cell line. Seven days after the implantation, mice were intravenously injected with a single 2 mg/kg dose of FF-10850 and TPT for five consecutive days, as indicated by arrows. The levels of human γH2AX in tumor (**A**) and mouse γH2AX in bone marrow (**B**) were monitored to assess DNA damage. Data are presented as mean ± standard deviation (*n* = 3 for FF-10850 and TPT groups, *n* = 2 or 3 for the vehicle control group).

γH2AX is a widely used marker for the detection of DNA double-strand breaks [33–35]. H2AX is rapidly phosphorylated upon DNA damage [36] and its phosphorylation level is directly correlated with the number of DNA double-strand breaks [37, 38]. Accordingly, we investigated the kinetics of γH2AX as a reflection of the distinct unencapsulated topotecan pharmacokinetic profiles in the tumor and bone marrow after administering FF-10850 and TPT. In the pharmacodynamic study, mice were administered a single intravenous injection of 2 mg/kg FF-10850, whereas TPT was injected for 5 consecutive days. The upregulation of human γH2AX levels was observed in the tumor after a single dose of FF-10850, exhibiting an intensity and duration similar to that observed after 5 consecutive doses of TPT (Fig. 4A). In the bone marrow, the increase in the mouse γH2AX levels was marginal in the FF-10850 group, whereas a notable increase was observed after each TPT dose (Fig. 4B). These findings suggest that FF-10850 affords a superior therapeutic index compared to TPT.

### PK/PD-based Dosing Schedule Improvement of FF-10850

**Fig. 5:**
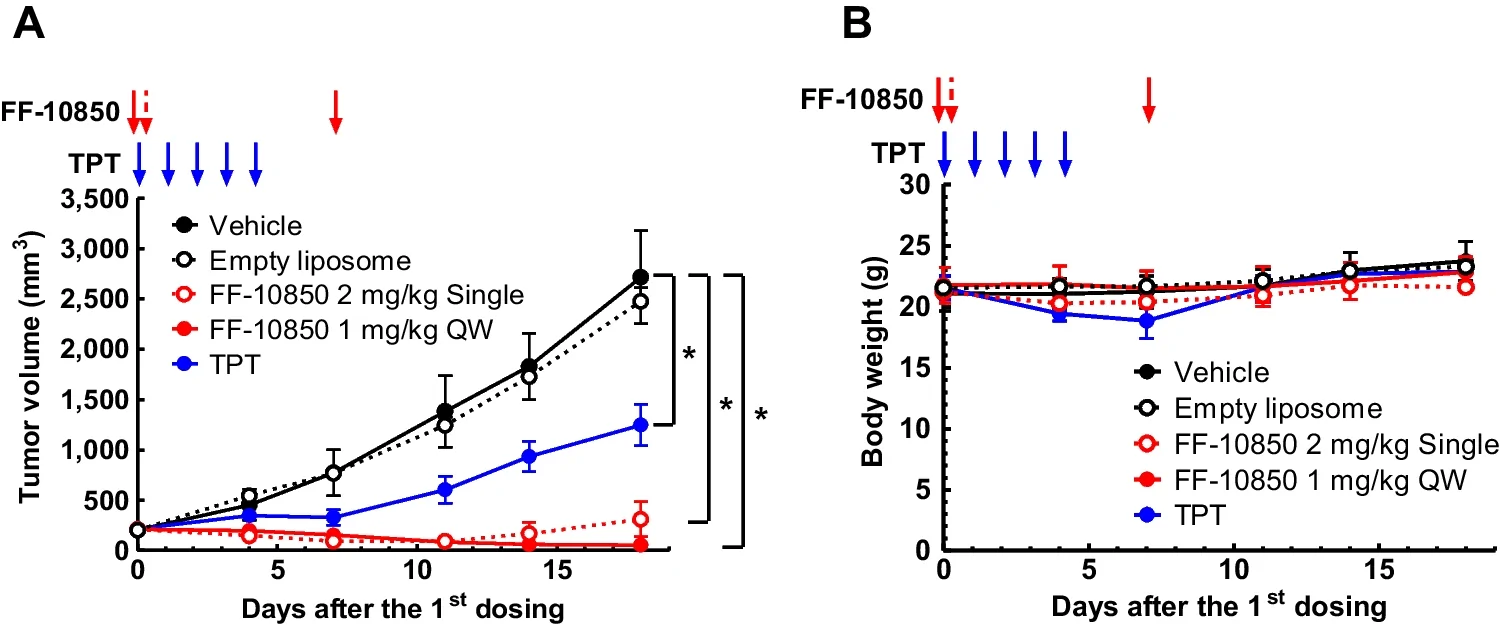
Antitumor activity of FF-10850 and TPT treatment. ES-2 xenograft model mice were intravenously administered with 2 mg/kg of FF-10850 once (Single) or with 1 mg/kg/week once weekly (QW) for 2 weeks, and 2 mg/kg of TPT for five consecutive days as indicated by arrows. As controls, sucrose/histidine buffer and empty liposome were intravenously administered once a week. Tumor volume (**A**) and body weight (**B**) were monitored. Data are presented as mean ± standard deviation (*n* = 8). Statistical analysis was performed using Bartlett’s test followed by Steel–Dwass. *p < 0.01.

Given the close relationship between the unencapsulated topotecan tissue distribution, γH2AX kinetics, and previously reported pharmacological responses (Fig. 3, 4 and [27]), we then explored potential improvements in the dosing schedule of FF-10850 to maximize its antitumor efficacy based on the PK/PD relationship. Considering the tumor kinetics of unencapsulated topotecan characterized by a peak concentration (C_max_) on the first-day post-dosing, followed by a gradual decrease and a return to baseline γH2AX levels from days 3 to 11 post-dosing (Fig. 3B and 4A), we hypothesized that weekly dosing would be required to sustain the tumor growth inhibitory effect of FF-10850. ES-2 tumor bearing mice were injected with FF-10850 once at a dose of 2 mg/kg or once a week for 2 weeks at a dose of 1 mg/kg with the same total dosage amount. Both FF-10850 treatment groups exhibited a significant tumor reduction when compared with the control group. Tumor regrowth was observed after 14 days post-dosing in the single administration group (Fig. 5A). Importantly, no severe body weight loss was observed in either of the FF-10850 groups (Fig. 5B). These outcomes align with our expectations, considering the rapid decrease in the unencapsulated topotecan concentration and the return of γH2AX steady-state levels in the bone marrow after 5 days of FF-10850 administration (Fig. 3C and 4B).

## Discussion

In the current study, we determined the pharmacokinetics and pharmacodynamics of the liposomal formulation FF-10850 compared to non-liposomal topotecan, aiming to understand the differences in tissue behavior underlying the therapeutic effects and safety profiles. Our findings demonstrate that encapsulation of topotecan in FF-10850 altered the tissue pharmacokinetics of unencapsulated topotecan, eliciting pharmacodynamic responses consistent with its better therapeutic effects than non-liposomal topotecan.

Analyzing the total exposure of topotecan, we observed that FF-10850 enhanced the accumulation of total topotecan in tumor tissue, potentially mediated via the EPR effect. In addition, despite the milder hematological toxicity, we unexpectedly observed elevated and sustained concentrations of total topotecan in the BM-ISF following FF-10850 administration (Fig. 1B, Table I). The bone marrow sinusoids have relatively large fenestrae that enable the passage of blood cells and proteins [39], probably contributing to the trafficking of FF-10850. The favorable therapeutic index of FF-10850 could not be solely explained by total topotecan exposure. Therefore, we focused on tissue exposure to unencapsulated topotecan and revealed that FF-10850 exhibited an improved ratio of unencapsulated topotecan exposure between the therapeutic target (tumor) and normal (bone marrow) tissues (Table II) in contrast to total topotecan exposure ratio. This improvement was consistent with its preferable pharmacological effects when compared with TPT (Fig. 4, 5, and [27]).

One of the notable advantages of liposome encapsulation of cytotoxic chemotherapeutic agents is the improved therapeutic index via the modification of their pharmacokinetics and biodistribution. In addition to circulation stability and tissue entry, the release of the unencapsulated drug and its subsequent tissue clearance govern the biological activities of liposomal drugs *in vivo*. Although tissue exposure to the bioavailable unencapsulated drug should correlate to the therapeutic and toxic effects of liposomal drugs, few reports have directly investigated the unencapsulated drug concentration in the target tissues [40]. Clinical studies with Doxil/Caelyx® have shown reduced cardiotoxicity, a serious and potentially life-threatening side effect of non-liposomal doxorubicin, by minimizing cardiac exposure [41, 42]. In both preclinical and clinical studies, pegylated liposomal doxorubicin was found to exert preferential accumulation in tumor tissues when compared with that in normal tissues [43–45]. The total doxorubicin concentration of Doxil/Caelyx® in pleural effusions of patients with breast, ovarian, and lung cancer was substantially higher than that of non-liposomal doxorubicin [8]. Furthermore, Doxil/Caelyx® has been shown to release its payload in tumor tissues partially, given that doxorubicin metabolites were detected in pleural effusion cells and supernatant [8]. However, its efficacy in patients with breast cancer was not superior to non-liposomal doxorubicin in a phase 3 clinical trial [10], suggesting that insufficient release efficiency in tumor tissue and/or entrapment of released doxorubicin in the lysosomal compartment of tumor cells diminishes its cytotoxic activity [46]. Similarly, ONIVYDE® (irinotecan liposome injection) has been estimated to provide less than 10% of total irinotecan available as unencapsulated irinotecan in tumor, based on a pharmacokinetic model developed from the distribution profile of total irinotecan and its metabolite, SN-38 [47]. The majority of the liposomal drug could be distributed as a biologically unavailable encapsulated form in tissues. These examples highlight the importance of measuring the unencapsulated drug, rather than the total drug, to understand the pharmacokinetic mechanisms underlying the pharmacological outcomes of liposomal drugs.

Despite the importance of pharmacokinetic information regarding unencapsulated drugs, the direct quantification of the unencapsulated drug in target tissues is challenging. Mechanical crushing of frozen tissue is commonly used to prepare the tissue homogenate; however, the freezing–thawing and/or excessive mechanical stress potentially disrupts liposomes and results in overestimation of unencapsulated drug concentration. For liposomes encapsulating prodrug forms such as ONIVYDE®, local tissue exposure to the active drug is measurable as an active metabolite [47, 48]. Microdialysis can also be used to measure the intratumoral unbound drug concentration but is associated with several limitations, such as recovery rates, difficulty with hydrophobic compounds, and the requirement of a sensitive detection method [49]. Moreover, the placement of microdialysis catheters into tissues is invasive and potentially affects local drug distribution. In the current study, we developed a non-freezing homogenizing method to separate unencapsulated topotecan from tissue homogenates with minimal mechanical stress. However, as non-freezing is involved, this method requires immediate sample processing at the site of harvest. Another feature of this method is its potential applicability to other liposomal drugs. Although our method was specifically developed for our liposomal formulation FF-10850, the method could be applied for evaluating unencapsulated drug exposure to other liposomal drugs. Furthermore, the development of next-generation liposomal drugs with controlled release mechanisms, such as thermosensitive or pH-sensitive mechanisms, is underway to address the limitations of the existing liposomal drugs [50]. Understanding the release kinetics in target tissues will be crucial for designing next-generation liposomal drugs.

Using our method for quantification of unencapsulated topotecan, we observed that FF-10850 elicited a lower C_max_ in BM-ISF and sustained exposure in the tumor to unencapsulated topotecan (Table II). Previous research conducted in a mouse model with OVCAR-3 xenograft revealed severe toxicities when topotecan was administered for five consecutive days. However, the highest efficacy was achieved following 20 daily injections at the same total dosage without any serious toxicity [51]. The authors suggested that maintaining plasma topotecan concentrations above the effective concentration for tumor cells is critical for antitumor activity, while peak plasma topotecan levels may contribute to hematological toxicity and subsequent mouse death. These findings suggest that reducing the maximum drug concentration in the bone marrow while prolonging the tumor exposure duration above the minimal effective drug concentration may enhance the therapeutic window. This supports the superior pharmacological effects of FF-10850 when compared to those of TPT, which induces greater DNA damage responses with transient but higher bioavailable topotecan concentrations in the bone marrow. Furthermore, FF-10850 improved the ratio of unencapsulated topotecan exposure between the therapeutic target and normal tissues (Table II), enhancing the safety margin. This improvement may be attributed to the accelerated payload release facilitated by the tumor microenvironment, characterized by high ammonia concentration and tumor-resident phagocytes, as suggested by a previous study [27]. In the present study, we observed that almost 100% of the payload was released within 24 h in tumor interstitial fluid (T-ISF) (Fig. S1), suggesting efficient topotecan release in extracellular space in tumor tissue.

In the bone marrow, TPT induced a sharp transient upregulation of γH2AX levels after each dose (Fig. 4B), indicating a rapid topotecan distribution and elimination (Fig. 3C). In contrast, FF-10850 induced a lower γH2AX upregulation during the dosing period (Fig. 4B). These findings are consistent with the unencapsulated topotecan exposure in BM-ISF observed in the current study and previous study [27], which demonstrated that FF-10850 mitigated hematological toxicity when compared with TPT. Although topotecan was rapidly cleared from tumor tissue similar to BM-ISF following TPT injection, γH2AX levels remained elevated for at least 24 h after each dose, suggesting possible retardation of DNA repair in tumor cells and apoptosis. In the tumor tissues of FF-10850-treated mice, we detected elevated γH2AX levels from the dosing day to 3 days post-dosing, followed by a gradual reversion to baseline until day 11 (Fig. 4A). Based on tumor γH2AX kinetics, we expected that FF-10850 would be more effective when dosed weekly than dosed once every 2 weeks at the same total dosage, which was confirmed in an efficacy study (Fig. 5). To optimize the dosing schedule for other tumor types and liposomal drugs, it would be valuable to construct a PK/PD model incorporating the cell killing kinetics linked with tissue pharmacokinetics, fine-tuned by the anti-tumor activity specific to each payload drug.

Our work highlights the importance of quantifying unencapsulated drugs in target tissues for the research and development of nanocarrier-mediated drug delivery systems. Although several challenges remain unresolved, the quantification method developed in the present study for unencapsulated drugs could be applicable to clinical research. Constructing a PK/PD model incorporating clinical pharmacokinetics of tissue unencapsulated drugs will enable rational dosing and prediction of pharmacological outcomes for liposomal anticancer pharmaceuticals. The tumor microenvironment, which influences the EPR effect and payload release mechanisms, has been recognized as a key factor for the effectiveness of nanocarrier-mediated antitumor therapeutics. However, it varies greatly among tumor types, individuals, and metastasis within the same patient [52]. The tissue pharmacokinetic information will assist in identifying biomarkers associated with drug accumulation and release, enabling patient stratification and optimization of the dosing regimen for liposomal pharmaceuticals.

## Conclusion

In the present study, we successfully developed a method to quantify unencapsulated topotecan in tumor tissue and BM-ISF following the administration of FF-10850. Our findings reveal that the tumor-to-BM-ISF ratio of AUC_inf_ for unencapsulated topotecan after FF-10850 administration is higher than that observed with non-liposomal TPT. This disparity may account for the superior pharmacological effects of FF-10850. Therefore, quantifying the exposure to the unencapsulated drug in therapeutic and toxic target tissues can provide a rationale for selecting the release strategy, designing the formulation, and determining the dosing regimen throughout the development and clinical research of liposomal drugs.

## Supplementary Information

Below is the link to the electronic supplementary material.

Supplementary file1 (DOCX 52.0 KB)

## Data Availability

All data analyzed in this study are fully disclosed and presented in detail within this article. Any additional raw data required to reproduce these findings can be provided upon reasonable request to the corresponding author.
