# Correction: Quantification of Unencapsulated Drug in Target Tissues Demonstrates Pharmacological Properties and Therapeutic Effects of Liposomal Topotecan (FF-10850)

**DOI:** 10.1007/s11095-026-04193-2

**Published:** 2026-10-08

**Authors:** Toshifumi Kimura, Ken Okada, Yasushi Morohashi, Shun Matsuda, Yukio Kato, Mikinaga Mori, Hiroshi Kato, Takeshi Matsumoto, Susumu Shimoyama

**Affiliations:** 1 Bio Science & Engineering Laboratories, FUJIFILM Corporation, Ashigarakami-Gun, 577 Ushijima, Kaisei-Machi, Kanagawa, 258-8577 Japan; 2 Safety Evaluation Center, Ecology & Quality Management Division, CSR Division, FUJIFILM Corporation, 210 Nakanuma, Minamiashigara, Kanagawa 250-0193 Japan; 3 Faculty of Pharmacy, Kanazawa University, Kakuma-Machi, Kanazawa, Ishikawa, 920-1192 Japan; 4 FUJIFILM Pharmaceuticals U.S.A., Inc, One Broadway, Cambridge, MA 02142 USA

**Correction: Pharmaceutical Research (2024) 41:795–806**

**https://doi.org/10.1007/s11095-023-03652-4**

The original article has been updated to include Dr. Shun Matsuda to be listed as co-author of this paper and to update the author contribution statement.

Published author list: Toshifumi Kimura· Ken Okada· Yasushi Morohashi· Yukio Kato· Mikinaga Mori· Hiroshi Kato· Takeshi Matsumoto· Susumu Shimoyama

New author list: Toshifumi Kimura· Ken Okada· Yasushi Morohashi· Shun Matsuda· Yukio Kato· Mikinaga Mori· Hiroshi Kato· Takeshi Matsumoto· Susumu Shimoyama

Published Author Contributions:

Conception or design of the work: Toshifumi Kimura, Mikinaga Mori and Susumu Shimoyama; Acquisition, analysis, or interpretation of data: Toshifumi Kimura, Ken Okada, Yasushi Morohashi, Yukio Kato, Hiroshi Kato and Takeshi Matsumoto; Drafting the work or revising it critically for important intellectual content: all authors; Final approval of the version to be published: all authors; Agreement to be accountable for all aspects of the work: all authors.

New Author Contributions

Conception or design of the work: Toshifumi Kimura, Mikinaga Mori and Susumu Shimoyama; Acquisition, analysis, or interpretation of data: Toshifumi Kimura, Ken Okada, Yasushi Morohashi, Shun Matsuda, Yukio Kato, Hiroshi Kato and Takeshi Matsumoto; Drafting the work or revising it critically for important intellectual content: all authors; Final approval of the version to be published: all authors; Agreement to be accountable for all aspects of the work: all authors.

