# Supplementary material for: Quantification of Unencapsulated Drug in Target Tissues Demonstrates Pharmacological Properties and Therapeutic Effects of Liposomal Topotecan (FF-10850)
Source: Pharm Res. 2024 Mar 27;41(4):795–806. doi: 10.1007/s11095-023-03652-4 (PMC11024016; doi:10.1007/s11095-023-03652-4)
Supplement: Supplementary file 1 — Supplementary file1 (DOCX 52.0 KB) [file 11095_2023_3652_MOESM1_ESM.docx]

**Quantification of unencapsulated drug in target tissues demonstrates pharmacological properties and therapeutic effects of liposomal topotecan (FF-10850)**

Toshifumi Kimura^1^, Ken Okada^1^, Yasushi Morohashi^1^, Yukio Kato^2^, Mikinaga Mori^1^, Hiroshi Kato^1^, Takeshi Matsumoto^1^, Susumu Shimoyama^3^

^1^ Bio Science & Engineering Laboratories, FUJIFILM Corporation, 577 Ushijima, Kaisei-machi, Ashigarakami-gun, Kanagawa 258-8577, Japan

^2^ Faculty of Pharmacy, Kanazawa University, Kakuma-machi, Kanazawa, Ishikawa 920-1192, Japan

^3^ FUJIFILM Pharmaceuticals U.S.A., Inc., One Broadway, Cambridge, Massachusetts 02142, USA

**Supplementary** **Table**

**Table S1 Lower limit of quantification in pharmacokinetic study**

|  |  | Total topotecan | Unencapsulated topotecan |
| --- | --- | --- | --- |
| Plasma | ng/mL | 0.625 | 0.500 |
| Tumor | ng/g tissue | 2.00 | 4.00 |
| BM-ISF | ng/mL | 13.7 | 13.7 |

BM-ISF, bone marrow interstitial fluid

**Supplementary** **Figure**

**Figure S1. Topotecan release in tumor interstitial fluid**

ES-2 tumor tissues freshly harvested from untreated mice were cut into small pieces on a membrane filter and placed in a centrifuge tube. The fluid obtained by centrifugation at 400 × *g* for 10 min at 4°C was collected as T-ISF. T-ISF was spiked with 4,000 ng/mL of FF-10850 and incubated for 0, 2, 7, and 24 h at 37°C. For separation of unencapsulated topotecan, samples were diluted with phosphate-buffered saline and ultracentrifugation at 200,000 × *g* for 1.5 h at 4°C. The supernatants were collected as samples for unencapsulated topotecan and quantified by LC-MS/MS. The percentage of unencapsulated topotecan was determined by total and unencapsulated topotecan concentrations at each time point. Data are presented as mean ± standard deviation (*n* = 3).
